# Orocutaneous Fistula Formation in Free Flap Reconstruction for Oral Squamous Cell Carcinoma

**DOI:** 10.3389/fonc.2022.887118

**Published:** 2022-04-26

**Authors:** Qigen Fang, Junhui Yuan, Wei Du, Liyuan Dai, Xu Zhang, Ruihua Luo

**Affiliations:** ^1^ Department of Head Neck and Thyroid, The Affiliated Cancer Hospital of Zhengzhou University & Henan Cancer Hospital, Zhengzhou, China; ^2^ Department of Radiology, The Affiliated Cancer Hospital of Zhengzhou University & Henan Cancer Hospital, Zhengzhou, China

**Keywords:** oral squamous cell carcinoma, orocutaneous fistula, free flap, risk factor, cachexia

## Abstract

**Objective:**

The aim of this study is to identify the risk factors associated with orocutaneous fistula (OCF) formation after free flap reconstruction for oral squamous cell carcinoma (SCC).

**Methods:**

Patients undergoing free flap reconstruction for oral SCC were retrospectively enrolled. The relationship between clinicopathologic variables and OCF formation was analyzed by univariate and multivariate analyses.

**Results:**

A total of 87 OCFs occurred in 856 patients. Univariate analysis revealed cachexia, tumor at the tongue/floor of the mouth (TFOM), T4 stage, preoperative hemoglobin level, pull-through procedure, preoperative albumin level, and surgical site infection were associated with the formation of OCF. Multivariate analysis confirmed the independence of cachexia, TFOM, T4 stage, and surgical site infection in predicting OCF development. Conventional wound care could achieve successful fistula closure in 82.4% of the patients with a median time of 28 days.

**Conclusions:**

OCF formation was common after free flap reconstruction. The presence of cachexia, TFOM tumor site, T4 stage, and surgical site infection significantly increased the risk of OCF formation. Although it required a long period, conventional wound care can obtain satisfactory outcomes in OCF management.

## Introduction

Oral squamous cell carcinoma (SCC) is the most common malignancy among head and neck cancers; it is characterized by local invasion and lymph node metastasis. Most of these tumors present in advanced stages at initial diagnosis (1, 2). Surgery is the preferred therapeutic approach, and defects caused by surgical excision are usually repaired using free flaps (3).

Complications after free flap reconstruction are frequently observed (4). An example would be an orocutaneous fistula (OCF). It can significantly delay the initiation of adjuvant therapies, increase the duration of a patient’s admission and costs of treatment, delay oral feeding and voice rehabilitation, and increase the risk of carotid blowout (5–7). Several prophylaxis and management procedures have been introduced. These consist of conventional wound care, negative pressure wound therapy, and surgical management (8–11), each having its corresponding indications. Moreover, numerous scholars have explored the predictors of fistula development in head and neck surgery. The risk factors comprise age, unhealthy lifestyle, systemic disease, tumor stage, previous radiotherapy, nutrition status, tumor site, and treatment method (12). However, these findings were concluded based on SCC in larynx and hypopharynx, which are significantly different from oral SCC.

To our best knowledge, there are only two studies on the predictors of OCF formation. Dawson et al. (13) enrolled 102 patients with different etiologies, such as cancer, osteoradionecrosis, ameloblastoma, and trauma for head and neck surgery, and found that OCF occurred in 11% of the patients. The only statistically significant association identified was between the formation of an OCF and previous chemoradiotherapy. Girkar et al. (14) reported a rate of 9% based on 587 eligible oral SCC patients, and the presence of surgical site infection and performing bilateral neck dissection posed the maximum risk for developing OCF.

Therefore, this current study aims to identify the risk factors associated with OCF formation after free flap reconstruction for oral SCC.

## Patients and Methods

### Ethical Considerations

The Institutional Research Committee approved our study, and all participants signed an informed consent agreement. All methods were performed in accordance with the relevant guidelines and regulations. All procedures performed in studies involving human participants were in accordance with the ethical standards of the institutional and/or national research committee and with the 1964 Helsinki Declaration and its later amendments or comparable ethical standards.

### Patient Selection

Medical records of adult (≥18 years) patients between January 2011 and January 2021 with surgically treated oral SCC were retrospectively reviewed. Enrolled patients must meet the following criteria: the disease was primary and a free flap was performed for the initial operation. Patients with a history of other malignancies were excluded. Information regarding demography, smoking, drinking, preoperative hemoglobin and albumin, systemic disease (hypertension, diabetes mellitus, cardiovascular, and cerebrovascular disease), cachexia, pathology, TNM stage according to the 8^th^ AJCC classification, treatment, postoperative complication, and fistula management was extracted and analyzed.

### Definitions of Variables

A smoker was defined as one who had smoked at least 20 cigarettes per day for at least 5 years. A drinker was defined as one who had drunk wine at least once per day for at least 5 years (15). The normal range of hemoglobin was 130–145 g/L for men and 115–150 g/L for women. The normal range of albumin was 35–55 g/L. Cachexia was defined as weight loss greater than 5% or weight loss greater than 2% in individuals already showing depletion according to current body weight and height or skeletal muscle mass (16). Surgical site infection (SSI) was defined as there was presence of purulent drainage from the wound or abnormal redness, swelling, pain, and so on of the wound (17, 18).

### Treatment

All operations were performed with lower lip splitting under general anesthesia. The tumor was resected with a margin of at least 1 cm, then the defect was reconstructed by a free flap, and neck dissection was performed for all patients. The muscles in the floor of the mouth (pull-through procedure) were resected if necessary. The postoperative flap was observed hourly during the first 24 h and then every 4 h for the next 3 days. All patients received cephalosporin as prophylaxis 30 min before incision, which was continued for at least 3–5 days after surgery. Nasogastric nutrition was routinely administered until patients were able to feed orally (at least 7 days) (19). Postoperative adjuvant radio-/chemoradiotherapy was given if there were adverse pathologic features.

### Orocutaneous Fistula

OCF referred to a fistula connecting the oral cavity and skin, and was clinically detected according to a combination of signs consisting of a foul odor emanating from the oral cavity, a visible breach in the intraoral suture line, collection in the neck, turbid drain, congestion and/or edema of the neck flap, fever, and leukocytosis (13, 14), and also verified by a swallow study or dye test. Conventional wound care including wound flushing with saline and hydrogen peroxide as well as compressive dressings was the first choice; negative pressure wound therapy and secondary surgery were selectively considered if the fistula was too large or the closure time was too long.

### Statistical Analysis

The association between clinicopathologic variables and OCF formation was first evaluated by the Chi-square test, then the significant factors were analyzed using multivariate analysis (binomial logistic regression). All statistical analyses were performed using SPSS 20.0, and *p* < 0.05 was considered significant.

## Results

A total of 856 patients were included, including 590 men and 266 women. The median age was 49 (range: 22–74) years. There were 573 smokers and 377 drinkers. Preoperative low hemoglobin and albumin levels occurred in 204 and 146 patients, respectively. A total of 234, 100, and 88 patients had hypertension, diabetes mellitus, and cardiovascular and cerebrovascular disease, respectively. Cachexia was noted in 378 patients (**Table 1**).

**Table 1:** Clinical and pathologic features in the 856 patients.

Variable	Number (%)
Age	
<49	428 (50.0%)
≥49	428 (50.0%)
Sex	
Male	590 (68.9%)
Female	266 (31.1%)
Smoker	573 (66.9%)
Drinker	377 (33.1%)
Preoperative hemoglobin level	
Low	204 (23.8%)
Normal	652 (76.2%)
Preoperative albumin level	
Low	146 (17.1%)
Normal	710 (82.9%)
Hypertension	234 (27.3%)
Diabetes mellitus	100 (11.7%)
Cardiovascular and cerebrovascular disease	88 (10.3%)
Cachexia	378 (44.2%)
Primary site	
Tongue/the floor of the mouth	455 (53.2%)
Buccal	234 (27.3%)
Gingiva	167 (19.5%)
Tumor stage	
T2	279 (32.6%)
T3	444 (51.9%)
T4	133 (15.5%)
Neck dissection	
Unilateral	702 (82.0%)
Bilateral	154 (18.0%)
Flap type	
Radial free forearm flap	386 (45.0%)
Anterolateral thigh flap	300 (35.0%)
Free fibula flap	170 (20.0%)
Pull-through procedure	237 (27.7%)
Flap failure	25 (2.9%)
Surgical site infection	199 (23.2%)
Orocutaneous fistula	87 (10.2%)

The distribution of the primary sites was as follows: tongue/floor of the mouth (TFOM) in 455 patients, buccal in 234, and lower gingiva in 167. For the tumor stages, T2 in 279 patients, T3 in 444, and T4 in 133. Unilateral and bilateral neck dissection was performed in 702 and 154 patients, respectively. There were 386 radial free forearm flaps, 300 anterolateral thigh flaps, and 170 free fibula flaps. The muscles in the floor of the mouth (pull-through procedure) were resected in 134 patients.

SSI occurred in 199 patients. Flap re-exploration was required in 90 patients, and 65 were successfully salvaged. Twenty-five flaps were lost, and among these patients, 20 underwent an immediate pectoralis major myocutaneous flap reconstruction, and five received direct suture. The overall success rate of free flap reconstruction was 97.1%.

### Association Between Clinicopathologic Variables and OCF Formation

OCF occurred in 87 patients, with an incidence of 10.2%. In 652 patients with normal preoperative hemoglobin levels, the incidence of OCF formation was 8.7%, which was significantly lower than the 14.7% incidence in patients with low preoperative hemoglobin levels (*p* = 0.014). In 710 patients with normal preoperative albumin levels, the incidence of OCF formation was 8.9%, which was significantly lower than the 16.4% incidence in patients with low preoperative albumin levels (*p* = 0.006). There was a significant difference (*p* < 0.001) in the incidence of OCF formation between patients with and without cachexia at 15.3% and 6.1%, respectively. Tumors at the TFOM had an OCF development rate of 13.2%, which was significantly higher than that in tumors located in the buccal mucosa or gingiva (*p* < 0.001). Among all tumor stages, T4 tumors had the highest rate of OCF development (*p* < 0.001). OCF occurred in 33 of the 237 patients requiring a pull-through procedure; the rate was significantly higher than that in patients without a pull-through procedure (*p* = 0.024). Of the 199 patients with SSI, 20.1% had OCF, which was significantly higher than 7.1% in patients without SSI (*p* < 0.001) (**Table 2**).

**Table 2:** Univariate analysis of the association between clinicopathologic variables and orocutaneous fistula (OCF) development.

Variable	OCF		*p*
	Yes (*n* = 87)	No (*n* = 769)	
Age			
<49	40	388	
≥49	47	381	0.428
Sex			
Male	65	525	
Female	22	244	0.218
Smoker			
No	25	258	
Yes	62	511	0.366
Drinker			
No	43	436	
Yes	44	333	0.195
Preoperative hemoglobin level			
Low	30	174	
Normal	57	595	0.014
Preoperative albumin level			
Low	24	122	
Normal	63	647	0.006
Hypertension			
No	60	562	
Yes	27	207	0.414
Diabetes mellitus			
No	72	684	
Yes	15	85	0.089
Cardiovascular and cerebrovascular disease			
No	79	689	
Yes	8	80	0.725
Cachexia			
No	29	449	
Yes	58	320	<0.001
Primary site			
Tongue/the floor of the mouth	60	395	
Buccal	2	232	
Gingiva	15	152	<0.001
Tumor stage			
T2	14	265	
T3	30	414	
T4	43	90	<0.001
Neck dissection			
Unilateral	70	632	
Bilateral	17	137	0.691
Flap type			
Radial free forearm flap	41	345	
Anterolateral thigh flap	35	265	
Free fibula flap	11	159	0.185
Pull-through procedure			
No	54	565	
Yes	33	204	0.024
Flap failure			
No	84	747	
Yes	3	22	1.000
Surgical site infection			
No	47	610	
Yes	40	159	<0.001

The multivariate analyses confirmed the independence of cachexia (*p* < 0.001, 2.816 [1.225–3.932]), TFOM site (*p* < 0.001, 1.998 [1.022–2.564]), T4 stage (*p* = 0.005, 2.316 [1.543–4.026]), pull-through procedure (*p* < 0.001, 4.336 [1.883–9.037]), and SSI (*p* < 0.001, 1.773 [1.028–2.886]) in increasing the risk of OCF development (**Table 3**).

**Table 3:** Multivariate analysis of the association between clinicopathologic variables and orocutaneous fistula (OCF) development.

Variable	*p*	OR [95% CI]
Preoperative hemoglobin level	0.111	1.887 [0.893–2.231]
Preoperative albumin level	0.326	2.001 [0.776–4.328]
Cachexia	<0.001	2.816 [1.225–3.932]
Primary site		
Buccal		
Lower gingiva	0.263	1.547 [0.847–3.143]
Tongue/the floor of the mouth	<0.001	1.998 [1.022–2.564]
Tumor stage		
T2		
T3	0.067	1.778 [0.965–2.795]
T4	0.005	2.316 [1.543–4.026]
Pull-through procedure	<0.001	4.336 [1.883–9.037]
Surgical site infection	<0.001	1.773 [1.028–2.886]

### Treatment of OCF

A total of 75 OCFs received conventional wound care only, the mean size of the OCF was 2.5 ± 1.7 cm, and all of them achieved full recovery. The median duration of OCF management was 28 (range: 3–63) days.

In the remaining 12 OCFs, the mean size was 3.5 ± 1.3 cm. Conventional wound care was also administered first, but did not demonstrate a significant benefit in fistula improvement after a mean duration of 40 ± 7.6 days. These patients finally received secondary operation using local flaps, and secondary OCF did not occur.

## Discussion

To our knowledge, this study included the largest sample size for analyzing the risk factors of OCF formation after oral SCC surgery. OCF formation after free flap reconstruction was common. The presence of cachexia, tumor site at the TFOM, T4 stage, and SSI significantly increased the risk of OCF. Although it requires a long period, conventional wound care can obtain satisfactory outcomes in OCF management.

Cachexia is related to weight loss, inadequate food intake, inactivity, loss of muscle mass, and metabolic derangement in patients with advanced cancer (16). It could result in more toxicities, poorer disease-free survival, and more treatment gaps (20). More importantly, postoperative complications are associated with cachexia. Among 2,531 patients undergoing hepatectomy, Lee et al. (21) reportedly had a higher occurrence of bleeding, infection, wound complication, and respiratory failure in the malnutrition cohort. A similar finding was also confirmed in other solid malignancies (22), but there are only a few reports regarding head and neck cancers. Hayashi et al. (23) enrolled 192 patients receiving chemoradiotherapy for head and neck cancer, and found that in a setting of chemoradiotherapy, all adverse events were strongly associated with cachexia. Among grade 3–4 adverse events, the frequency of leukopenia, anemia, and neutropenia had significant differences between patients with and without cachexia. The current study may be the first to identify the profound significance of cachexia in causing OCF formation; cachexia impaired wound healing, causing fistula formation. This finding carries meaningful clinical importance as it indicates that greater attention is required for cachexia patients to prevent OCF development.

The primary site, tumor stage, and pull-through procedure were significantly associated and also predictive factors. Due to the special location of TFOM in the oral cavity, treatment of these advanced-stage tumors often required mandibular lip-split surgery or pull-through resection, and extensive excision usually resulted in the creation of dead space. Al Deek et al. (6) emphasized the importance of dead space obliteration in preventing OCF occurrence. The dead space between the mandible and hyoid bone was originally occupied by extrinsic tongue muscles, and should have been filled with viable tissue during reconstruction. The part of the flap stuffed in the dead space should be checked for reliability after establishing perfusion, especially when the excess muscle was resected or its orientation was changed during the inset of the flap. Our findings also supported this viewpoint. Both primary TFOM site and T4 stage were associated with higher rates of OCF formation. Similarly, Girkar et al. (14) reported OCF development in approximately 15% of patients with TFOM SCC compared to 7% in patients with SCC in other subsites, irrespective of the type of flap used. Additionally, even if early oral intake is not allowed, motion of the reconstructed TFOM still occurs during the recovery period. If these movements occur after a non-ideal design, they could impair the water-tight closure leading to saliva accumulation at the TFOM as this is a dependent part of the oral cavity. Thus, tension must be distributed by multilayer mattress sutures to minimize dehiscence and suture line tearing.

SSI was also a factor that could not be ignored. It occurred in up to 15%-45% of the patients undergoing surgery for head and neck SCC (17, 18, 24). SSI was the result of the interaction of multiple elements, such as prolonged surgical duration, faulty surgical techniques, poor oral hygiene, poor nutritional status, type of reconstruction, and resection range. The negative effects of these factors on incision healing was confirmed in pharyngeal cutaneous fistula (4). Generally, fistula and infections coexist, resulting in a vicious cycle where one may lead to the other. Consistent with the finding of Girkar et al. (14), the current study clarified the effect of SSI on OCF formation.

Other researchers have reported the predictive value of bilateral neck dissection and previous chemoradiotherapy (13, 14). The association between neck dissection and OCF formation may be explained by increased blood loss and increased duration of surgery. Moreover, bilateral neck dissection was only performed if the primary tumors extended across the midline or if there were pathologically proven lymph nodes in the contralateral neck, indicating an extensive disease. However, the current study failed to note this positive relationship. A difference in the study design might have contributed to this variation. Previous chemoradiotherapy significantly posed an apparent effect on wound healing and tissue fibrosis (25). This dysfunction could explain the tendency of OCF formation in patients with previous chemoradiotherapy.

The treatment of OCF is often time-consuming, usually requiring 4 weeks or longer (5). Currently, there are three kinds of management for OCF, conventional wound care, negative pressure wound therapy, and surgical intervention (6–11). Numerous studies have demonstrated the reliability of negative pressure wound therapy. Tian et al. (8) reported successful closure in 90% of OCFs *via* this method with a median time of 19 days. Inatomi et al. (10) also reported that 82.4% of fistulas were addressed by this method, with a mean closure time of 30.4 days. A recent review by Khoo et al. (5) confirmed the superiority of negative pressure wound therapy over conventional wound care by reducing the closure time, but with a similar success rate. Secondary surgery was usually the last choice after conservative treatment. Our findings also showed that conventional wound care was achieved at a success rate of 86.2% using local flap for the residual cases.

Limitations in the current study must be acknowledged. First, it was retrospective, thus possessing inherent bias. Second, we did not calculate the effect of OCF on oncologic survival. Third, routine lower lip splitting was debatable and may increase additional risk of OCF occurrence.

In summary, OCF formation after free flap reconstruction was common. The presence of cachexia, tumor at the TFOM site, T4 stage, and SSI significantly increased the risk of OCF formation. Although it required a long period, conventional wound care could obtain satisfactory outcomes in OCF management.

## Data Availability Statement

The original contributions presented in the study are included in the article/supplementary material. Further inquiries can be directed to the corresponding author.

## Ethics Statement

The Institutional Research Committee of Henan Cancer Hospital approved our study, and all participants signed an informed consent agreement. The patients/participants provided their written informed consent to participate in this study.

## Author Contributions

All the authors made a contribution in study design, manuscript writing, study selection, data analysis, study quality evaluation, and manuscript revision. All authors have read and approved the final manuscript.

## Conflict of Interest

The authors declare that the research was conducted in the absence of any commercial or financial relationships that could be construed as a potential conflict of interest.

## Publisher’s Note

All claims expressed in this article are solely those of the authors and do not necessarily represent those of their affiliated organizations, or those of the publisher, the editors and the reviewers. Any product that may be evaluated in this article, or claim that may be made by its manufacturer, is not guaranteed or endorsed by the publisher.
